# Exploring regulatory mechanisms on miRNAs and their implications in inflammation-related diseases

**DOI:** 10.1007/s10238-024-01334-y

**Published:** 2024-07-03

**Authors:** Emre Nalbant, Yeliz Z. Akkaya-Ulum

**Affiliations:** Department of Medical Biology, Faculty of Medicine, Hacettepe University, 06100 Sihhiye, Ankara Türkiye

**Keywords:** microRNAs, Cellular regulation, Epigenetic modifications, Inflammatory conditions, Therapeutic interventions

## Abstract

This comprehensive exploration delves into the pivotal role of microRNAs (miRNAs) within the intricate tapestry of cellular regulation. As potent orchestrators of gene expression, miRNAs exhibit diverse functions in cellular processes, extending their influence from the nucleus to the cytoplasm. The complex journey of miRNA biogenesis, involving transcription, processing, and integration into the RNA-induced silencing complex, showcases their versatility. In the cytoplasm, mature miRNAs finely tune cellular functions by modulating target mRNA expression, while their reach extends into the nucleus, influencing transcriptional regulation and epigenetic modifications. Dysregulation of miRNAs becomes apparent in various pathologies, such as cancer, autoimmune diseases, and inflammatory conditions. The adaptability of miRNAs to environmental signals, interactions with transcription factors, and involvement in intricate regulatory networks underscore their significance. DNA methylation and histone modifications adds depth to understanding the dynamic regulation of miRNAs. Mechanisms like competition with RNA-binding proteins, sponging, and the control of miRNA levels through degradation and editing contribute to this complex regulation process. In this review, we mainly focus on how dysregulation of miRNA expression can be related with skin-related autoimmune and autoinflammatory diseases, arthritis, cardiovascular diseases, inflammatory bowel disease, autoimmune and autoinflammatory diseases, and neurodegenerative disorders. We also emphasize the multifaceted roles of miRNAs, urging continued research to unravel their complexities. The mechanisms governing miRNA functions promise advancements in therapeutic interventions and enhanced insights into cellular dynamics in health and disease.

## Introduction

The flow of information from DNA to protein, famously named the “Central Dogma” includes numerous regulation steps involving countless biomolecules. This regulation is performed at many levels, including DNA accessibility, expression of RNAs and proteins, along with post-transcriptional and post-translational control of stability and half-life [1]. Still, these systems of regulation may not respond fast enough to coordinate every cell function to adapt to endogenous or exogenous stimuli. Therefore, another mechanism of regulation involves synthesis of non-coding RNAs, especially, small non-coding RNAs. Their small size, diversity, and quantity inside the cell make these molecules perfect regulators of “fine-tuning control.” They can modulate gene expression levels, block DNA binding sites, prevent mRNA translation, increase diversity of splicing products, help protect cells from foreign DNA and RNAs, and temporally modulate protein synthesis [2]. Non-coding RNAs’ ability to epigenetically modify and differentiate cells and their reactions to the environment, highlights their role in protecting the thin line between healthy and diseased states of organisms.

Among the non-coding RNAs, microRNAs (miRNAs) are classified as short non-coding RNA with a length of around 22 nucleotides. 4630 experimentally validated miRNAs have been identified according to the miRTarbase update 2022 [3]. More than 60% of the human protein-coding genes have complementary sites for miRNAs. The epigenetic roles of miRNAs are classically described as mRNA repression resulting in decreased translation of the gene through these complementary sites. Besides this canonical pathway, other non-canonical roles for miRNAs have been defined, as explained in the following sections.

miRNAs’ epigenetic and messenger roles are mostly studied regarding cancer pathologies but their systemic effects in inflammatory and immune diseases are increasingly drawing attention. Variations in circulating or cellular miRNA expression and epigenetic functions in relation to inflammatory disease pathogenesis have been described in many studies [4, 5]. In this review, we provide a summary of miRNA biogenesis, mechanisms regulating miRNA expression and their mechanism implications in various inflammatory diseases.

## miRNA biogenesis

**Fig. 1:**
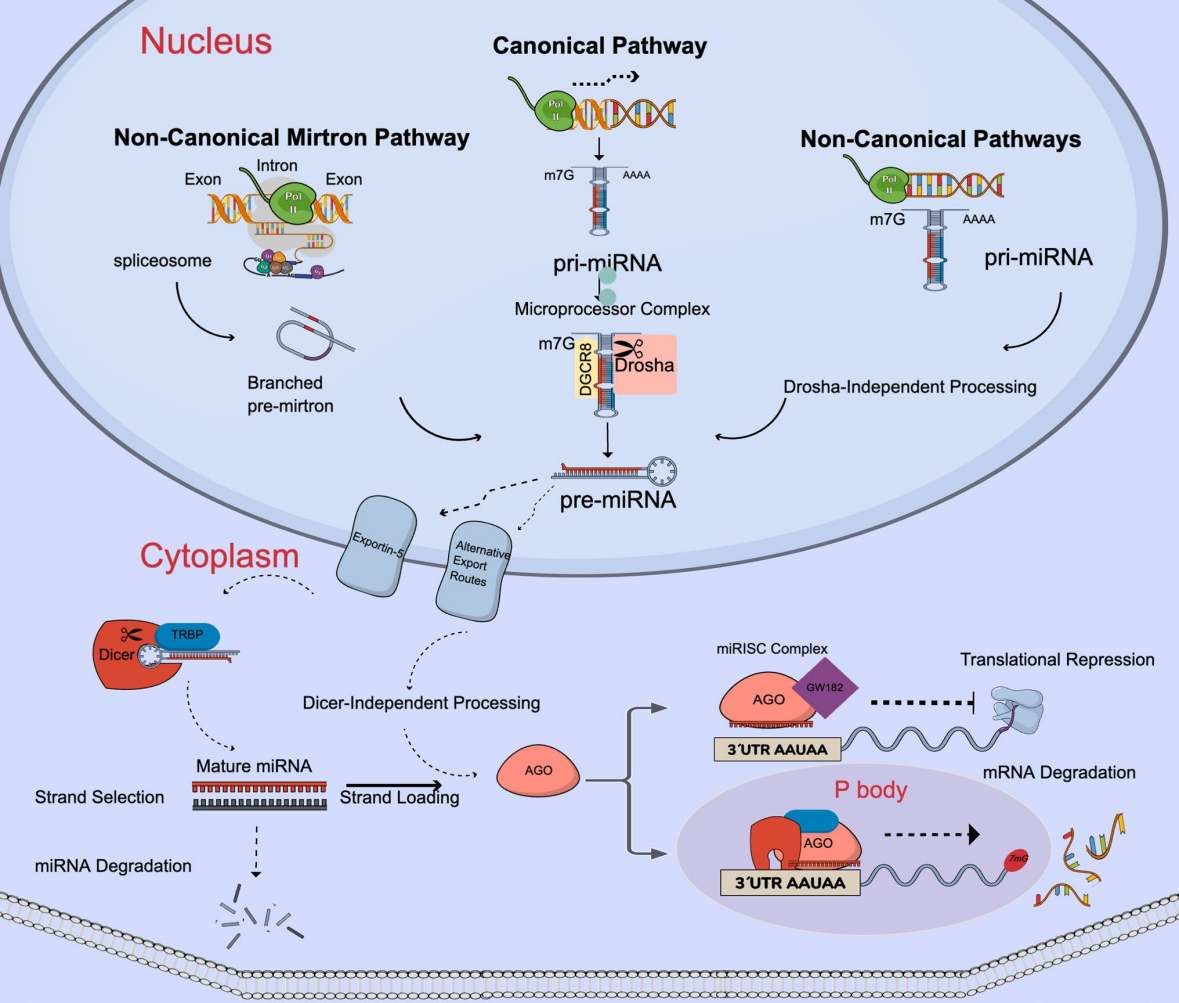
miRNA biogenesis and mechanism of action. Canonical miRNA biogenesis begins with RNA polymerase II enzyme transcribing from the miRNA gene or miRNA gene clusters. pri-miRNAs are 5′ capped and poly-A tail attached RNA. Microprocessor complex consists of Drosha and DGCR8, further process hairpin structures to generate a single hairpin RNA molecule called as pre-miRNA. Pre-miRNA is exported to the cytoplasm via Exportin-5. Inside the cytoplasm pre-miRNA interacts with the Dicer and TRBP and loses its hairpin structure. Non-canonical mirtron pathway produce miRNAs from the introns of the coding genes. Introns discarded from splicing of mRNA, form a lariat structure, and from this branched pre-mirtron pre-miRNA is produced. Pre-miRNAs leave the nucleus through Exportin-5 or alternative export routes. Drosha-independent and Dicer-independent pathways also generate mature miRNAs but their biogenesis mechanism is still not well-understood. Pre-miRNA processing leaves double-stranded short RNA molecule and from this either the 5p or the 3p strand is selected and loaded on to the AGO family member proteins to form the miRISC complex. miRISC complex interacts with the mRNA 3′UTR site according to the seed sequence of miRNA and with the help of GW182 protein suppresses translation by effecting ribozyme. GW182 interacts with the RNA-degrading enzymes to degrade mRNA after translational repression. Alternatively, miRISC complex inside P-bodies directly degrades mRNAs without temporal silencing. Figure created in the Mind the Graph platform, www.mindthegraph.com

Long pri-miRNA sequences distributed in the genome can be found in intronic or exonic sites of coding sequences or as clusters coding hundreds of miRNAs with their own promoter [2]. All these miRNA sequences are transcribed by RNA polymerase II enzyme and later undergo splicing, which widely enhances their diversity and number. Transcribed pri-miRNAs are capped, and tailed, and can hold hundreds of miRNA sequences. After their synthesis, pri-miRNAs form hairpin structures, these secondary structures expose the base sequences unique for recognition of nuclear microprocessor complex. This complex comprises the RNase III enzymes Drosha ribonuclease III (DROSHA) and DiGeorge syndrome chromosomal region (DGCR), which work together to cut every hairpin structure from long pri-miRNA to produce single hairpins called pre-miRNAs which are ~ 55 to 70 nucleotides long. Transcribed pri-miRNAs during their shortening inside the nucleus are the targets for RNA modification enzymes [6]. Specific methylations, base exchange, and phosphorylation of nucleotides can occur. Characteristic processing by the DROSHA enzyme leaves pre-miRNA with 3ʹ hydroxyl group overhangs of 2 nucleotides and a 5ʹphosphate group, which is another recognition signal for Exportin-5, an export receptor found in the nucleus. Pre-miRNAs are carried to the cytosol for further shortening to expose the “seed sequence” and binding to the partner protein. Dicer protein captures the pre-miRNA with the help of dsRBP trans-activation-responsive RNA-binding protein (TRBP) and cuts the pre-miRNA hairpin to generate double-stranded RNA that is 20–25 nucleotides. Pri-miRNA hairpin formations and Dicer cutting generate two miRNA sequences called -5p and -3p according to their location. Both RNAs have the potential to be mature miRNA, and both contain 5′ seed sequences. Transportation of the miRNA double-stranded structure to the Argonaute RISC component (AGO) protein depends on ATP and Hsc70-Hsp90 chaperones [7]. AGO protein while choosing the guide strand controls for the 5′ nucleotide sequence patterns of both complementary miRNAs and their relative thermostability [8]. Selecting one strand causes a change in AGO structure and the other strand leaves the complex to degradation (Fig. 1). DROSHA, Dicer, and Exportin-5 seem indispensable proteins for miRNA biogenesis, but Young-Kook et al. knocked out these three genes individually in the same human cell lines and observed that the miRNA production process was disrupted but not destroyed. DROSHA knock-out led to extremely low miRNA expression, but synthesis of some miRNAs continued [9]. This led to the identification of a DROSHA-independent branch of non-canonical miRNA biogenesis (Fig. 1). Other non-canonical pathways like Ago2-slicing dependent pathways, Dicer-independent pathways, and different export routes are uncommon in general miRNA biogenesis, but necessary to produce specific miRNAs. Protein coding genes intron sites may generate microRNAs named mirtrons, this type of RNA produces miRNAs without the need for a microprocessor, as an example of a non-canonical pathway [10] (Fig. 1).

## miRNA action

Mature miRNAs are ready for their functional role following their proper alignment within the m microRNA-induced silencing complex (miRISC). Ago proteins possess four functional domains that hold and carry the miRNA. AGO2 protein is the major protein for the miRISC complex but other AGO proteins involvement has been seen in different organisms and a variety of cell types. miRNA localization into AGO protein simultaneously happens with Dicer cutting and choosing the sense strand of pri-miRNA. The cutting pattern of Dicer allows the miRNA recognition by AGO protein and strand loading occurs in terms of the seed sequence. This silencing complex also requires helper proteins like GW182. GW182 protein domains bind to the AGO complex, stabilize mRNA, and recruit other enzymes. In terms of mRNA and RISC complex stability, GW182 is an essential protein. The canonical function of the RISC complex starts with 2–7 nucleotide matching of seed sequence with mRNA, binding with the mRNA causes repression of translation. After the repression of translation, the types of RNA-binding proteins (RBPs) recruited to the complex decide the fate of the mRNA. Deadenylating proteins recruited via GW182 shorten the poly-A tail, whereas decapping enzymes facilitate the removal of the protective 5′ cap, allowing endonucleases access for the degradation of mRNAs. These processes mostly occur in processing bodies (P-bodies). P-bodies are the sites enriched with degradation enzymes, proteins’ proximity with each other increases the efficiency of the degradation processes. RISC complex abundance is high in P-bodies, but they are also distributed through the cytoplasm to search the target mRNAs.

miRNAs, unlike other non-coding RNAs (e.g., small interfering RNAs (siRNAs)), do not require perfect complementarity with their targets. While seed region matching is important for silencing, other regions of miRNA may also match with the mRNA. The main recognition site is the 3′ UTR of the mRNAs but miRNA matching also occurs in exonic regions and 5′ UTRs of the mRNA. mRNA sequences include reoccurring or different matching sites for miRISC complexes. Co-operative binding of miRISC complexes to multiple sites within one mRNA increases the likelihood for its repression. Another regulatory factor for the binding and function of miRISC is other RBPs and their localization sites on mRNA. Depending on the RBPs interaction and proximity to the miRISC complex on a mRNA molecule, their effect can be antagonist or synergetic. RISC complex interactions with the mRNA interrupt the translation of mRNA, and depending on the RBPs and helper protein interactions suppression of translation can be temporary or can lead to degradation of mRNA.

miRNA binding to mRNA does not lead to repression of the mRNA every time, positive regulation of transcription and translation can be seen. miR-10 targeting 5′ UTR of ribosomal protein mRNAs under amino acid scarcity conditions increases the translation of proteins and is one of the examples of a dual role of miRNAs [11]. This transcriptional activation mechanism has been observed under cellular stress and has not been mechanistically identified yet.

Apart from miRNAs functions in the cytoplasm, miRNAs and miRISC complexes have also been shown to be localized in the nucleus. Mature miRNA transportation into the nucleus mostly depends on the hexanucleotide sequence (AGUGUU) found in the 3′ UTR site of the miRNAs according to studies on miR-296 [12]. Many miRNAs’ abundances in the nucleus are low compared to their cytoplasmic localization but some miRNAs like miR-25 and miR-29a have a higher number in the nucleus. These abundance differences between cytoplasm and nucleus are tissue-specific, differentiation and division-related and can be stress-related [13]. Nuclear transportation of mature miRNAs is important for their further miRNA editing processes and their functional roles in the transcription. Nucleolar body localization of miRNAs can reflect their function, such as miRNA’s effect on rRNA synthesis found in nucleolus with other related proteins affecting the rRNA transcription. According to Roberts et al., miRNAs localized in the nucleus can regulate the stability of the nuclear transcription, join and regulate alternative splicing during transcription, and induce epigenetic modification as seen in siRNA-mediated gene silencing [12].

The canonical role of miRNA is suppression of mRNA molecules but their seed sequence matching with the transcription site or binding of the long non-coding RNAs (lncRNAs) close to the gene promoter may change transcription efficiency through steric effect. The interaction between small nuclear RNAs (snRNAs) and miRISC complex affects the splicing patterns of pre-mRNAs. Another important role for these miRNAs is transcriptional gene silencing (TGS) and transcriptional gene activation (TGA), nucleotide match between different regulator sequences and miRNAs affect epigenetic modifications that regulate transcription. In terms of transcriptional activation, blocking the repressor sequences causes activation of the gene. The interactions between AGO proteins and histone modifiers can generate more stable epigenetic marks on gene loci, this can result in acetylation or methylations depending on the complex histone modifications [14]. Korla et al. propose another mechanism for nuclear miRNAs, miRNA’s interaction with transcription factors may guide the TFs into their target site and the following computational matching results consolidate this hypothesis [15].

From the nucleus to the cytoplasm, miRNAs affect the chromatin remodeling, transcription, splicing, and translation efficiency of mRNAs. Their imperfect matching strategy allows them to activate or silence related pathways in a time-dependent manner to change cellular dynamics in terms of environmental change.

Because of miRNA compartmentalization in extracellular vesicles for their protection, miRNA’s function is not restricted to the cellular levels so they can show their effect on the systemic level [16]. Their varying abundance in circulation or release to the extracellular matrix changes the cellular dynamics of other cells.

## Regulation of miRNA expression level

**Fig. 2:**
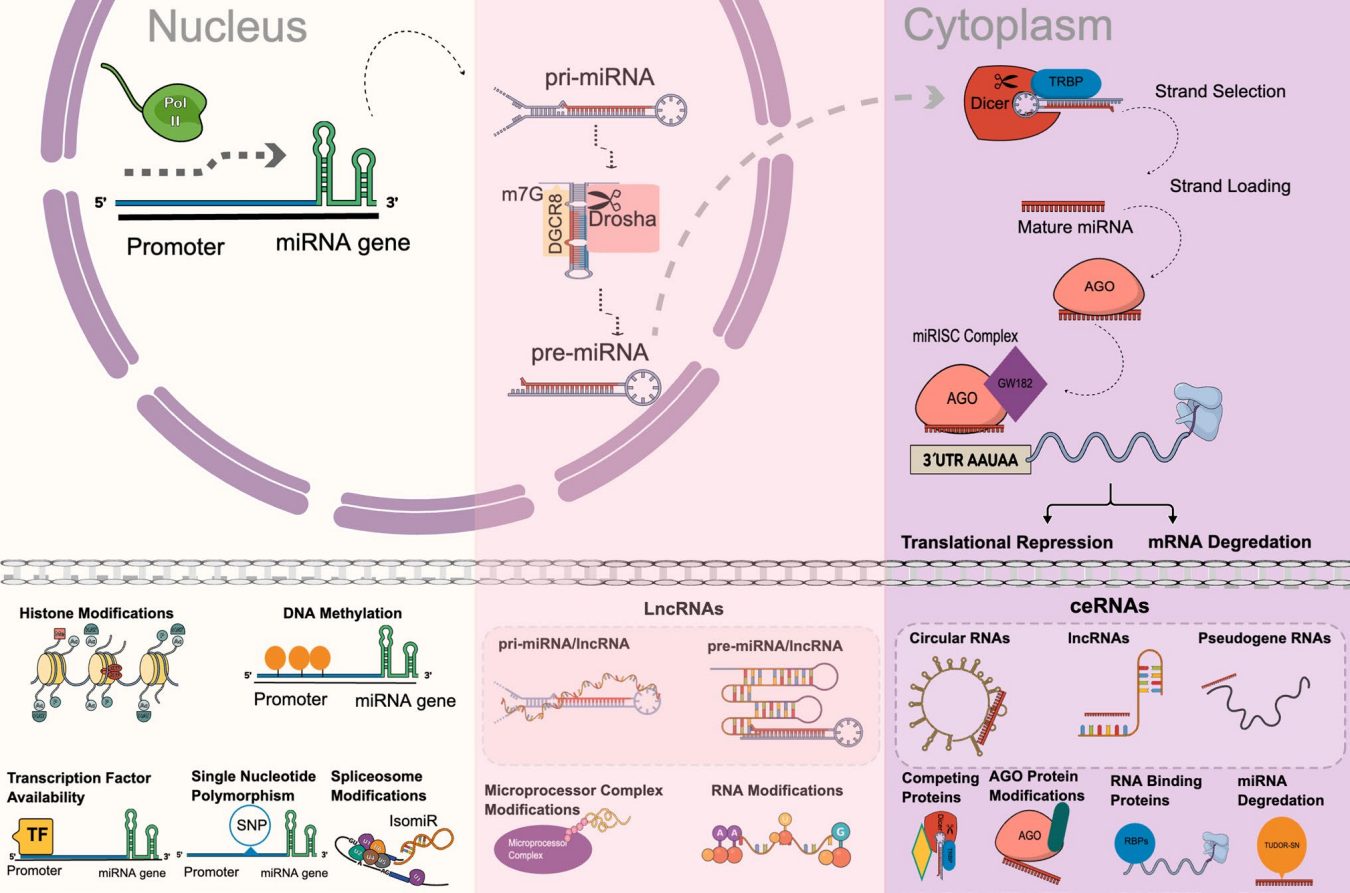
Epigenetic factors affecting miRNA biogenesis and function. There are five main mechanisms related with miRNA genes in nucleus: DNA methylation, histone modifications, and the availability of transcription factors, SNPs and spliceosome modifications. SNPs found in miRNA genes and regulatory elements affect the stability and synthesis of pri-miRNA. Spliceosome modifications can change the miRNA gene product and generate isomiRs of one miRNA. There are three mechanisms related with pri-miRNAs and pre-miRNAs regulation in nucleus: 1—They are affected by the microprocessor protein’s function and modulation. 2—lncRNAs localize in the nucleus interact with the pri/pre-miRNAs and effect their export. 3—RNA modifications on pri/pre-miRNAs can change the half-life and further processing steps of miRNA. There are five main pathways for post-transcriptional regulation of miRNAs in cytoplasm: Dicer protein interactions with helper, competing proteins, AGO protein modifications, ceRNAs and RNA-binding proteins. AGO protein modifications change the mature and functional miRNA number inside the cell. ceRNAs like circular RNAs, lncRNAs and pseudogene RNAs with their complementary sequences can sponge and lead them to degradation. RNA-binding proteins regulate the function of the miRISC complex and miRNA degradation controls the miRNA abundance in cytoplasm. Figure created in the Mind the Graph platform, www.mindthegraph.com

miRNAs’ expression levels are distinctively regulated within cells. Organisms in this regard control miRNA activity in every step of their lifespan and actively change their abundance inside the cells. There are many regulatory mechanisms like transcription factors, epigenetic regulation of miRNA expression, miRNA editing, miRNA sponges and RNA-binding proteins, and miRNA degradation that cause changes in miRNA expression level (Fig. 2).

### Transcription factors

miRNA transcription mostly depends on transcription factors (TF). The initial step in miRNA expression regulation is binding to miRNA promoter sites in the genome. Hypoxia-inducible factor one (HIF1), a well-known transcription factor regulating hypoxia, activates transcription of miR-210 for downstream regulations [17]. Inflammation-related NF-κB-mediated regulation of transcription drastically changes the miRNA expression profile, which can have diverse effects depending on the cell type [18]. E2F transcription factor 1 (E2F1) synthesis directly activates the miR-17-92 miRNA cluster which will repress the E2F1 translation forming a negative feedback loop, while the activation of specific miR-106 family miRNAs increases E2F1 transcription [19].

### Epigenetic regulation of miRNA expression

Exogenous or endogenous signals show different effects depending on the cell type and differentiation status. A fundamental explanation for this difference is epigenetic modifications such as DNA methylations and histone modifications during differentiation. miRNA sequences found inside coding genes’ exons or introns are affected by the hosting genes’ promotor methylation pattern. On the DNA level, methylation of cytosine residues prevents the binding of transcription factors and RNA polymerase enzymes. Methylation occurs on CpG islands, and it is found that miRNA sequences rich in CpG islands in their promoter region or intergenic regions are affected by methylation mostly. Cytosine methylation usually leads to the silencing of the gene and hypermethylation causing the loci to become inaccessible for interacting proteins and TFs, but alternatively, methylated regions may show an increase in splicing activity. Methylation in flanking regions of miRNA clusters affects RNA polymerase activity and transcripts are further processed by DROSHA or degraded by other enzymes. Glaich et al. found that heavy methylated miRNA regions have high DROSHA localization leading to splicing activity, removing the methylation marks on DNA caused decreased miRNA expression [20]. Deregulation of methylation and demethylation of miRNA loci independent of the differentiation status of the cell, are seen in many diseases, especially in cancer. miR-182 methylation status changes have been demonstrated in acute myeloid leukemia, triple-negative breast cancer, and sensory organ development-related diseases [21–23]. Some miRNAs repress or activate the DNA methyltransferase (DNMT) proteins’ activity resulting in DNA methylation profile changes of many genes and other miRNAs’ [24]. Regulations on methylating or demethylating enzymes have varying effects on miRNA synthesis and expression profile.

Histone protein modifications such as methylation and acetylation are well-known epigenetic regulation mechanisms. These modifications in histone proteins affect the histone-DNA interaction by changing their affinity to DNA leading to transcriptionally open or closed regions. They are regulated by histone modification enzymes like histone deacetylases (HDACs) or histone acetyltransferases (HATs), DNA hypermethylation sites, and non-coding RNAs. miRNAs with RISC complex can guide a change in these modifications, and miRNA synthesis depends on these factors. Dysregulated histone modification may open chromatin structures for aberrant miRNA synthesis or vice versa it can block the synthesis of necessary miRNAs. Abnormal miRNA levels in tissues seen in disease progression show a correlation with a dysregulated histone code [25].

### miRNA editing

Microprocessor complex proteins, transport proteins, and cytoplasmic processors of miRNAs are heavily regulated inside cells. Microprocessor proteins are affected by the non-coding RNAs due to their role in the splicing mechanism by choosing one type of miRNA from the same code and nucleolar proteins whose effects change the stability of the complex. Post-transcriptional regulation like splicing and miRNA editing are the regulators on the miRNA levels and mediator enzymes controlled by their levels and relative functions. They can affect miRNAs’ stability by binding, leading to increased expression of RNAs. The shortening of pri-miRNAs by DROSHA generates isomers of specific miRNAs that differ in length, sequence, or both. These isomers may have a variety of functions in the cell, but they can also be novel biomarkers for many diseases [26]. Dicer protein cutting function is evolutionary better preserved, but it can also generate isomers. Its relationship with the pre-miRNAs is controlled by other proteins an example of this precise regulation is the affinity race for Dicer domains. TARBP2 subunit of RISC loading complex (TARBP2) is an essential partner of Dicer, and RB binding protein 6 (RBBP6) protein competes for the same domain to interact with Dicer. Binding of one or the other leads to the differential processing of pre-miRNA [27, 28]. Editing of the RNA sequences is another modification method for regulation. miRNA editing enzymes include adenosine deaminase RNA specific protein (ADAR), which changes adenosine to inosine, and cDAR, which changes cytosine to uracil. Both edits change the target binding of the miRNA and modulate the stability of the RISC complex [29]. Non-templated adding is another editing method that has varying effects on miRNA function. Interestingly, Koppers-Lalic et al. observed that non-templated adding mechanisms differ for cellular and extracellular miRNAs, such that 3′ end uridylation is mostly found in miRNAs inside EVs and 3′ end adenylation in miRNAs of cellular origin [30]. Furthermore, miRNA polymorphisms can alter their synthesis at every step, causing a shift in the miRNA profiles in cells [31].

### miRNA sponges and RNA-binding proteins

RNA-binding proteins (RBPs) can compete with miRNAs to occupy the mRNA-binding sites only to protect the mRNA or interact with the RISC complex to free it from the mRNA. As discussed before, RBP localization onto mRNA, depending on its proximity to miRISC complex induces effective silencing and stabilizes the miRISC complex.

miRNA alterations inside cells also depend on RNA-RNA interactions. Long RNA transcripts like pseudogene RNAs, lncRNAs, and circular RNAs with their complementary sequences to miRNAs can sponge miRNAs to protect mRNAs. Their natural form is found in various organisms and due to their functions, they named as competing endogenous RNAs (ceRNAs). Single lncRNA or circular RNA can interact with different miRNAs and negatively regulate many different pathways simultaneously [32]. Some miRNAs are directly synthesized from long non-coding RNA genes and small nucleolar RNA (snoRNA) genes via splicing [33]. Transcribed pseudogene RNAs, lacking the sequence for translation, may act as decoy targets instead of the functional mRNA of the same gene. According to Ebert and Sharp, ceRNAs may decrease the abundance of miRNAs to inhibit their effect, or they can set a threshold for miRNA function. Tissue-specific expressed ceRNAs may increase the miRNA-RISC complex stability through helping the strand selection by neutralizing the passenger strand [34] Stable RNA structures like circular RNAs can capture miRNAs inside and outside of the cell [35].

### miRNA degradation

As discussed before, miRNA editing may change miRNA function, target, and stability but it can be used as a degradation mark in many organisms. Tudor staphylococcal nuclease (Tudor-SN) protein, a ubiquitously and evolutionary protected nuclease, degrades RNAs with extensive modifications specifically adenosine-to-inosine modifications and IU matching sites in hairpin structure [36]. Dicer protein interaction with ADAR proteins increases this mark on the miRNAs leading to a decrease in miRNA levels. Tudor-SN activity is limited to the modified miRNAs, but another mechanism named target-directed miRNA degradation (TDMD) causes specific degradation of miRNAs determined by their 3′ UTR sites in the RISC complex [37]. miRNA target interactions depend on imperfect matching with its target and matching algorithms group miRNA target interactions as 6-mer, and 7-mer by their paired sequences. Complete matching with its target changes its conformation thus leaving the 3′ UTR site unprotected inside AGO. TDMD proteins modify 3′ UTR sites of miRNA, trimming them and unloading from the AGO protein causing miRNA decay [38]. The sponging mechanism and ceRNA interactions may lead to TDMD-involved decay [39].

Non-templated adding can change the stability of the miRNA. Uridylation of miRNAs by terminal uridyl transferase (TUT) family proteins lead to degradation of the miRNA. One well-known example of complex regulation by the TUT family proteins is the differentiation-inducing let-7 miRNA suppression by Protein lin-28 homolog A(LIN28A) [40]. LIN28A binding to miRNA increases the number of uracil marks added by TUT4 or TUT7. Uracil labeling for degradation is an essential way of downregulating miRNA biogenesis, function and quantity in the cells.

Defects on this fundamental biogenesis and RISC complex-related proteins lead to non-canonical ways of miRNA expression and overall decrease the abundance of miRNAs. GW182 protein malfunctioning disturbs the stability of the RISC-mRNA-binding and related ribonuclease enzyme interactions, directly causing spontaneous miRNA degradation [39].

## Dysregulation of miRNA expression in human disease

Increasing focus on miRNA’s role in different human diseases demonstrated wide disturbances in miRNA levels change from tissue to tissue and in terms of disease pathology (Table 1). Cancer cells release miRNAs to their surroundings, manipulate neighboring cells to induce growth hormones and evade immune cells by inhibiting their differentiation followed by activation of inflammation. Even monogenic diseases with known mutation hide epigenetic mysteries under their pathology [41]. miRNA profile changes, in autoinflammatory and autoimmune diseases are under intense research due to their systemic effects, but less is known about why this profile changes (Table 1). In this section, our focus will be on elucidating the mechanisms underlying altered miRNA expression levels, which subsequently contribute to inflammation-related diseases.

**Table 1:** Inflammation-related diseases with altered miRNA expression levels and miRNA regulations

Inflammation-related diseases	Altered miRNA	Regulation mechanism on altered miRNA	References
Systemic sclerosis	Decreased miR-150	DNA methylation	[42]
Hidradenitis suppurativa	Changed miRNA profiles	Altered biogenesis machinery	[43]
		RISC complex	[44]
Psoriasis	Increased miR-21	LncRNA	[45]
	Decreased miR-26a		[46]
Vitiligo	Increased miR-25	DNA hypomethylation	[47]
	Decreased miR-211	LncRNA	[48]
Rheumatoid arthritis	Decreased miR-124a	DNA hypermethylation	[49]
	Decreased miR-22-3p	Histone modification	[50]
	Decreased miR-124a	Circular RNA	[51]
Osteoarthritis	Increased miR-29b	DNA hypomethylation	[52]
	Decreased miR-140-5p	DNA hypermethylation	[53]
	Decreased miR-146a		
	Decreased miR-195-5p	Circular RNA/DNA methylation	[54]
	Decreased miR-93	Circular RNA/DNA methylation	[55]
Atherosclerosis	Decreased miR-10a	DNA methylation	[56]
	Decreased miR-195-3p	Histone modifications	[57]
Crohn’s disease	Decreased miR-145	DNA hypermethylation	[58]
	Increased miR-21	DNA hypomethylation	[59]
Systemic lupus erythematosus	Decreased miR-1246	Histone modifications	[60]
	Decreased miR-142-3p/5p		[61, 62]
	Decreased miR-146		
	Decreased miR-125a-3p	Circular RNA	[63, 64]
	Increased miR-126	DNA hypomethylation	[65]
	Decreased miR-30d	Histone modifications	[66]
TRAPS	Decreased miR-146a	Degradation by IRE1-dependent cleavage	[67]
	Decreased miR-155		
Multiple sclerosis	Decreased miR-21	DNA methylation	[68]

### Skin-related autoimmune and autoinflammatory diseases

In the realm of skin-related autoimmune and autoinflammatory diseases, miRNAs emerge as a central player in orchestrating molecular processes. Disorders such as psoriasis and systemic lupus erythematosus showcase the intricate interplay between aberrant immune responses and miRNA regulation. The involvement of miRNA in skin pathology provides a compelling avenue for understanding the mechanisms driving these conditions and is essential for many different autoinflammatory diseases that have skin manifestations [43].

#### Systemic sclerosis

Systemic sclerosis is a rare multisystem autoimmune disease with involvement of inflammation and characterized by collagen-induced tissue fibrosis [69]. Honda N. and colleagues identified decreased mir-150 expression caused by DNA methylation in scleroderma fibroblast cells, causing increased integrin β3 expression and TGF-β signaling, ultimately inducing collagen gene transcription leading to fibrosis [42]. Hidradenitis suppurativa and psoriasis are common skin inflammatory diseases with similar pathogenetic mechanisms [44]. Hessam et al. searched for the miRNA machinery regulation in inflammatory lesions of hidradenitis suppurativa patients and found that compared to healthy skin, main components of miRNA processing machinery like Drosha, Dicer, Exportin-5 and DGRC8 were significantly altered [43]. The following year, same team of researchers found AGO and AGO-related protein levels dropped in the inflammatory sites; however, protein activator (PACT) levels remained the same in inflamed tissue [44]. These findings demonstrate the effect of inflammatory signals effect on the regulation of the miRNA machinery in the nucleus and cytoplasm.

#### Psoriasis

Psoriasis is a common inflammatory skin disorder that 3% of the world’s population suffers from. It involves the proliferation or aberrant differentiation of keratinocytes [45]. Examples of lncRNA effects on miRNAs can be seen in psoriasis studies. A study found that decreased lncRNA maternally expressed gene 3 (MEG3) expression in psoriatic cells lifted the ceRNA effect of MEG3 on miR-21 and increased levels of miR-21 to inhibit caspase 8 regulated apoptosis [45]. Another study on psoriasis in IL-22/LPS-stimulated HaCaT cells and psoriasis mice model showed another lncRNA effect, non-coding RNA activated by DNA damage (NORAD) lncRNA increase in cells decreased miR-26a and its inhibitory effects on cell proliferation [46]. Studying the epigenetic mechanism behind miRNA seems a promising way to find a cure.

#### Vitiligo

Vitiligo is a chronic autoimmune skin disease that targets the melanocytes, which miRNA expression profile differences seen between healthy parts of the tissue and disease parts. Metastasis associated lung adenocarcinoma transcript 1 (MALAT1) is lncRNA highly expressed in the nucleus and a negative upstream regulator of miR-211 which is a downregulated miRNA in lesional epidermis [48]. Another study identifies reactive oxygen species as a factor leading to the dysregulated expression of miRNAs in vitiligo. ROS-induced suppression of DNA methyltransferases (DNMTs) leads to hypomethylation of the miR-25 locus, causing more demethylation and suppression of melanocyte-inducing transcription factor [47].

### Arthritis

Recent research has spotlighted the role of miRNAs in the context of inflammatory and arthritic conditions. Unraveling the dynamic relationship between inflammation, arthritis, and miRNA holds promise for developing targeted therapeutic approaches for these complex health issues.

#### Rheumatoid arthritis

Rheumatoid arthritis (RA) is a multifactorial autoimmune disease affecting the joints and the most common type of arthritis [70]. Due to its complex nature, epigenetic regulations are thought of as the regulators of disease progression. Zhou and et al. found that a decrease in miR-124a in synovial tissues was caused by the DNA hypermethylation of the three + gene loci for miR-124a [49]. Following their experiments, they showed the positive effect of demethylating agent 5-AzadC on miR-124a expression and cell phenotype [71]. Histone methyltransferase enhancer of zeste 2 polycomb repressive complex 2 subunit (EZH2) also plays a role in RA pathogenesis by binding the promoter sites and bringing the H3K27me3 modification to the histone protein which suppresses miR-22-3p synthesis [50]. Circular RNA-HIPK3 (CircHIPK3) levels in RA patients correlate with the decrease in miR-124a [51]. In RA, many circular RNAs and miRNAs studied have the potential to be the biomarker of the disease state or the potential therapeutic target [72].

#### Osteoarthritis

Osteoarthritis (OA) is another well-studied arthritic disease. OA cartilage tissues show decreased DNA methyltransferase 3B (DNMT3B) and an increase of miR-29b decreased levels of DNMT3B cannot sustain the normal methylation levels on miR-29b promoter until IL-1β stimulation. DNMT3B induction upon stimulation increases the methylation on the promoter region of miR-29b causing the gene to be silent [52]. Another study on OA found hypermethylation on miR-140-5p and miR-146a, which decreased the binding affinity of SMAD family member 3 (SMAD-3) and NF-κB transcription factors for their activation [53]. CircFADS2’s protective role from LPS-induced cell injury is not seen in OA, whereas healthy chondrocytes after LPS stimulation increase CircFADS2 circular RNA levels, decrease miR-195-5p levels, and cause methylation on its promoter [54]. A related study found that increased circ_HECW2 levels in OA leads to increased apoptosis upon LPS stimulation and suppresses the protective effects of miR-93 by causing promoter methylation [55]. These two studies show that the opposing roles of miRNAs and lncRNAs on apoptosis.

### Cardiovascular diseases

In cardiovascular diseases, the impact of inflammatory processes is a significant aspect of pathophysiology. Inflammation plays a crucial role in the initiation and progression of various cardiovascular conditions. Understanding the intricate relationship between inflammation and cardiovascular health is essential for advancing targeted therapeutic strategies in the field.

#### Atherosclerosis

Atherosclerosis is a chronic inflammatory disease that underlies serious cardiovascular events, such as stroke and ischemic heart diseases. A genome methylation analysis study comparing atherosclerotic and healthy aorta samples identified hypermethylation on differentially methylated regions (DMRs) in chromosome nine open reading frame 3 (C9orf3) which contains six miRNA sites [73]. One of the studies on the variability of the methylation profiles in atherosclerosis patients found that the low-density lipoprotein cholesterol content and MIR10A gene cg06760035 site methylation showed a correlation with disease score [56]. Wang et al. studied the estrogen receptor α (ERα)’s protective effects in atherosclerosis and concluded that decreased miR-152 levels cannot sustain the control of DNMT1 mRNA and its translation [74]. The ERα promoter is under the control of DNMT1 hypermethylation. Homocysteine in circulation is an independent risk factor causing activation of macrophages, and in a 2021 study done with model mice, it was found that homocysteine (HcY) treatment led to miR-195 hypermethylation in macrophages. It is affecting the SP1 protein which controls the HDAC enzymes to change the chromatin marks on miR-195 [57]. HcY metabolite also changes the human vascular endothelial cells’ miRNA profile, miR-22-3p, and miR-1229-3p causing endothelial dysfunction [75]. HcY-induced changes create a domino effect for the progression of the chronic inflammatory disease.

### Inflammatory bowel disease

In maintaining a delicate balance between beneficial and harmful bacteria, the intestines play a pivotal role actively regulated by immune cells. The delicate balance between immune activation and tolerance is crucial for overall gut health. Perturbing this equilibrium, however, can lead to the development of inflammatory bowel disease (IBD). These disorders, encompassing conditions like Crohn’s disease and ulcerative colitis, manifest as chronic inflammation within the gastrointestinal tract.

#### Crohn’s disease

In Crohn’s disease, there is lifelong inflammation causing bowel damage. In Crohn’s disease, intestinal mucosa barrier dysfunction plays an important role. A team of scientists studying the mechanisms for increased transcription factor SOX-9 (SOX-9) expression in Crohn’s disease found a decrease in miR-145 levels due to a hypermethylated promoter site [58]. Genome-wide methylation analysis study found that the miR-21 gene hypomethylation was confirmed for the adult patients [59].

### Autoimmune and autoinflammatory diseases

Autoimmune and autoinflammatory diseases are characterized by dysregulated immune responses that lead to the body’s immune system attacking its own cells and tissues. In autoimmune diseases, the immune system mistakenly recognizes self-components as foreign, initiating an aberrant immune response and resulting in chronic inflammation. Conversely, autoinflammatory diseases are marked by unprovoked inflammation without a clear autoimmune component, causing periodic episodes of fever, pain, and organ damage as seen in systemic autoinflammatory disorders (SAIDs) patients [5].

In the context of autoimmune and autoinflammatory diseases, miRNAs called as inflammamiRs play pivotal roles in modulating gene expression, influencing immune cell function, and contributing to the dysregulation observed in these pathologies [1, 76].

#### Systemic lupus erythematosus

Systemic lupus erythematosus (SLE), a multifaceted autoimmune disorder, intricately involves the immune system mistakenly targeting various organs and tissues in the body. This systemic autoimmune disease often leads to chronic inflammation and a range of symptoms affecting the skin, joints, kidneys, and other organs [62]. In recent years, researchers have delved into the molecular intricacies of lupus, uncovering the significant role played by miRNAs in its pathogenesis [77]. miRNA-126 is an intronic miRNA found in *EGFL7* gene and it targets the DNA (cytosine-5)-methyltransferase 1 (DNMT1) protein. Inverse correlation between miR-126 and DNMT1 seen in SLE and RA [65, 78]. Hypomethylation of *EGFL7* gene promoter up-regulates the miR-126 which changes the methylation profile around other genes like CD11a and CD70 by decreasing the DNMTI1 protein levels. Abnormally active B cells are seen in SLE patients with decreased p53 expression and dysregulated miRNA profile, and Zhang et al. studied the mechanism of tumor protein 53 (p53) and mir-1246 interactions in B cells and found an increase in H3K27me3 and a decrease in H3K9/K14ac in the miR-1246 promoter site [60]. p53-induced activation of histone modification enzymes resulted in miR-1246 silencing and abnormal B cell activation. This type of abnormal activation can be observed in CD4 T cells of SLE patients. Ding Shu et al. tested the role of BCL-6 transcription repressor (BCL-6) on miR-142 downregulation seen in SLE, and their results showed that activated BCL-6 directly binds to a promoter-proximal element of mir-142 in healthy cells and in SLE T4 cells this binding is stronger [61]. This direct binding suppresses miR-142-3p/5p expression due to an increase in H3K27me3 levels and a reduction in H3K9/K14ac levels on the promoter site. Mycophenolic acid (MPA) treatment to CD4 T cells of the SLE patients and treatment decreased the silencing marks on histones while increasing the expression of miR-142 3p/5p and miR-146a compared to untreated CD4 cells of SLE but still decreased expression compared to healthy cells observed. This study identifies an important mechanism for MPA action in SLE treatment, compared to other therapeutic agents which had no effect on the miR-142 3p/5p and miR-146a levels [62]. Other than histone modifications circular RNAs play an active role in SLE. hsa_circ_0012919 which is increased in SLE patients significantly in lupus nephritis, leukopenia, anti-dsDNA ( +), C3 or C4 deficient types of SLE, and it is shown that it is a ceRNA for miR-125a-3p [63, 64]. Podocyte injury seen in Lupus nephritis can be another example of epigenetic regulations on miRNA such as TGF-B induction to human podocytes and kidney cells showed increased localization of Smad2/3 to miR-30d promoter and recruits the histone deacetylation (HDACs) enzymes to the site [66]. Dai R. et al. stated that in lupus-prone mice model splenic cells, hypomethylated genomic imprinted DLK1-Dio3 domain mostly contains miRNA transcripts related to lupus pathogenesis [79].

#### Behçet syndrome

Behçet disease is a complex and rare disorder characterized by a unique combination of autoimmune and autoinflammatory features. This condition involves abnormal inflammation of blood vessels, leading to a range of symptoms affecting various parts of the body [80]. As both an autoinflammatory and autoimmune disease, Behçet poses challenges in understanding its precise mechanisms. Although polymorphisms are not an epigenetic regulation mechanism, in Behçet disease, polymorphisms may explain the altered miRNA profiles. Such as miR-146a polymorphism rs2910164 causes a mismatch in the stem-loop of miRNA and changes its further modifications leading to its differential expression which can be seen as a protective polymorphism in Behçet disease and RA [81].

#### Tumor necrosis factor receptor-associated periodic syndrome

Tumor necrosis factor receptor-associated periodic syndrome (TRAPS) is a rare genetic autoinflammatory syndrome caused by mutations in tumor necrosis factor receptors [67]. Unfolded protein response activation seen in IL-1β stimulated TRAPS dermal fibroblast cells is due to the endoplasmic reticulum to nucleus signaling 1 (IRE1) and TNF receptor superfamily member 1A (TNFR1) relation and IRE1-dependent cleavage of miR-146a and miR-155 occurs [67].

### Neurodegenerative disorders

Neurodegenerative diseases are a group of disorders characterized by progressive degeneration of the structure and function of the nervous system. Emerging evidence suggests that miRNAs, small non-coding RNA molecules, play critical roles in the intricate regulatory networks underlying neurodegeneration. These miRNAs are involved in post-transcriptional regulation of gene expression, influencing processes such as protein aggregation, neuroinflammation, and neuronal survival [82].

#### Multiple sclerosis

Multiple sclerosis (MS) is one of the most common causes of neurological chronic inflammatory disease in young adults. Differences between miRNA profile and regulation due to common environmental factors in the relapsing–remitting phase of the disease studied by Ruhrmann et al. showed that methylation levels in the *MIR21* locus were higher in relapsing–remitting (RR-MS) patients and this methylation pattern followed a negative correlation with age [68].

## Discussion

miRNAs play a crucial role in the intricate regulatory networks governing cellular processes. Their small size, diversity, and ability to modulate gene expression make them powerful agents of fine-tuning control in various cellular functions. The biogenesis of miRNAs involves a complex series of steps, from transcription and processing in the nucleus to maturation and functional integration into the RISC in the cytoplasm. Non-canonical pathways and various regulatory proteins contribute to the versatility of miRNA biogenesis. The dysregulation of miRNAs is evident in various pathologies, particularly in cancer, autoimmune diseases, and inflammatory conditions. Understanding the epigenetic mechanisms and factors influencing miRNA abundance, such as DNA methylation and histone modifications, provides insights into the dynamic regulation of miRNAs. In this review, epigenetic regulations affecting miRNAs, which itself is an epigenetic factor and examples of dysregulation in human diseases are discussed. The well-known silencing role of miRNAs on mRNA is regulated immensely from their biogenesis in the nucleus to the degradation enzymes in the cytoplasm. Organisms control their response to environment and differential processes through changing miRNA profile but in diseases as seen in this article, it can act like a domino effect for the disease progression. Transcription factors, DNA methylations, and histone modifications affecting miRNAs’ expression are being controlled by other miRNAs creating an ambiguous picture about who affected who first, and miRNAs’ messenger role between cells propagates the altered miRNA expression. The interaction between miRNAs and transcription factors, as well as their sensitivity to environmental signals, emphasizes their adaptability and responsiveness to changing cellular conditions. Additionally, competition with RNA-binding proteins, sponging mechanisms, and affinity racing contribute to the complexity of miRNA-mediated regulatory networks. The degradation and editing of miRNAs further exemplify the intricate control mechanisms governing their levels and functions.

Inflammation and its high priority cause stress to many different cell types and as we discussed here different profiles seen in many tissues. The alterations in miRNA expression profiles observed in diseases like skin-related autoimmune and autoinflammatory diseases, arthritis, cardiovascular diseases, inflammatory bowel diseases, autoimmune and autoinflammatory diseases, and neurogenerative disorders highlight the potential of miRNAs as diagnostic markers and therapeutic targets. This enhances the importance of studying the miRNA regulations in diseased and healthy tissues. A growing number of reports suggest a significant utility of miRNAs and other small RNA drugs for clinical medicine [83]. Most miRNAs are attractive drug targets, but there are some specific challenges associated with miRNA therapy like off-target effect, effective dosage, or differential delivery methods, mainly caused by the short target’s sequence.

The multifaceted roles of miRNAs in cellular regulation, and their involvement in various diseases underscore the significance of continued research to unravel the complexities of these small yet powerful molecules. Understanding the nuanced mechanisms governing miRNA functions holds promise for advancing therapeutic interventions and enhancing our knowledge of cellular dynamics in health and disease.
